# The Productive Entry Pathway of HIV-1 in Macrophages Is Dependent on Endocytosis through Lipid Rafts Containing CD4

**DOI:** 10.1371/journal.pone.0086071

**Published:** 2014-01-22

**Authors:** Bonnie van Wilgenburg, Michael D. Moore, William S. James, Sally A. Cowley

**Affiliations:** Sir William Dunn School of Pathology, University of Oxford, Oxford, United Kingdom; Institut National de la Santé et de la Recherche Médicale, France

## Abstract

Macrophages constitute an important reservoir of HIV-1 infection, yet HIV-1 entry into these cells is poorly understood due to the difficulty in genetically manipulating primary macrophages. We developed an effective genetic approach to manipulate the sub-cellular distribution of CD4 in macrophages, and investigated how this affects the HIV-1 entry pathway. Pluripotent Stem Cells (PSC) were transduced with lentiviral vectors designed to manipulate CD4 location and were then differentiated into genetically modified macrophages. HIV-1 infection of these cells was assessed by performing assays that measure critical steps of the HIV-1 lifecycle (fusion, reverse transcription, and expression from HIV-1 integrants). Expression of LCK (which tethers CD4 to the surface of T cells, but is not normally expressed in macrophages) in PSC-macrophages effectively tethered CD4 at the cell surface, reducing its normal endocytic recycling route, and increasing surface CD4 expression 3-fold. This led to a significant increase in HIV-1 fusion and reverse transcription, but productive HIV-1 infection efficiency (as determined by reporter expression from DNA integrants) was unaffected. This implies that surface-tethering of CD4 sequesters HIV-1 into a pathway that is unproductive in macrophages. Secondly, to investigate the importance of lipid rafts (as detergent resistant membranes - DRM) in HIV-1 infection, we generated genetically modified PSC-macrophages that express CD4 mutants known to be excluded from DRM. These macrophages were significantly less able to support HIV-1 fusion, reverse-transcription and integration than engineered controls. Overall, these results support a model in which productive infection by HIV-1 in macrophages occurs via a CD4-raft-dependent endocytic uptake pathway.

## Introduction

Human macrophages are one of the main targets for HIV-1 infection, despite their moderately low surface expression levels of the main HIV-1 receptor, CD4 [1]–[6]. In macrophages, CD4 is constitutively recycled through clathrin-dependent endocytosis, a pathway from which it can also be subject to lysosomal degradation. Additionally, the presence of CD4 at the macrophage surface can be regulated by various biological and experimental stimuli [7]–[11]. The sub-cellular distribution of CD4 in macrophages differs from the other main HIV target cell type, T cells, due to T cell expression in T cells, but not macrophages, of Lymphocyte-specific protein tyrosine kinase (LCK), which is lipid raft-associated and retains CD4 at the plasma membrane [12]–[14]. It is currently not known whether the sub-cellular CD4 distribution in macrophages affects the ability of HIV-1 to enter through a successful pathway in these cells. However, the great majority of HIV-1 particles that bind to a susceptible cell fail to establish successful (‘productive’) infection, and this is particularly relevant in macrophages, which are relatively resistant to infection compared to T cells [15]–[17]. The distribution of CD4 in macrophages may influence the site of fusion of the virus envelope with a cell-limiting membrane, and therefore directly impact the efficiency of infection.

Previous pharmacological, biochemical and imaging studies have provided lines of evidence for an endosomal entry route for HIV-1 that is dependent on detergent resistant membranes (DRMs) [15], [16], [18], [19]. However, pharmacological agents may have multiple (off-target) or non-specific effects. Moreover, biochemical and imaging approaches cannot distinguish between productive and non-productive pathways. A genetic approach to studying the entry pathways in macrophages would enable more precise manipulation of key gene products; however, this approach has been lacking, due to the difficulty in genetically manipulating primary macrophages [18]. Myeloid cell lines, such as THP-1, can be genetically manipulated, but they do not faithfully recapitulate the properties of terminally differentiated macrophages and do not closely represent the HIV-1 entry pathway of macrophages [19]–[21]. To overcome these difficulties and extend the repertoire of experimental techniques available to investigate the productive entry pathway of HIV-1 in macrophages we have adopted a novel genetic approach. Using a technique previously developed in our laboratory, human macrophages can be derived simply and efficiently from human pluripotent stem cells (PSC), which are tractable to genetic modification using lentiviral vectors, as described previously [22]–[24]. PSC-macrophages are phenotypically and functionally similar to blood-derived macrophages and have a normal karyotype. Most importantly, they accurately replicate the HIV-1 replication kinetics seen in blood derived macrophages [22]–[24]. This cellular system by-passes the challenges posed by direct genetic manipulation of heterogeneous primary macrophages, namely low efficiency, variability and activation of the macrophages [25]–[31]. We have developed a lentivirus-based dual-expression system in order to genetically manipulate the PSC and study HIV-1 infection in the differentiated macrophages. This system enables the development of stably genetically engineered PSC lines, which can be used for a limitless number of experiments. The system permits simultaneous expression of transgenes, along with an shRNA to knock-down the expression of endogenous proteins. Using this system, we have expressed LCK in PSC-macrophages to stabilise CD4 surface expression. We have also knocked-down expression of wild-type CD4 while concomitantly expressing transgenes encoding shCD4-resistant, mutant versions of CD4, that localize outside DRMs. HIV-1 infection is measured at the most critical stages of the infection pathway - fusion, reverse transcription and expression from integrants. This has produced a novel set of evidence that supports a model whereby productive HIV-1 infection in macrophages requires CD4 localization to DRM and occurs via a DRM-dependent endocytic uptake pathway.

## Results

### Higher levels of CD4 are found in macrophages expressing LCK

**Figure 1:**
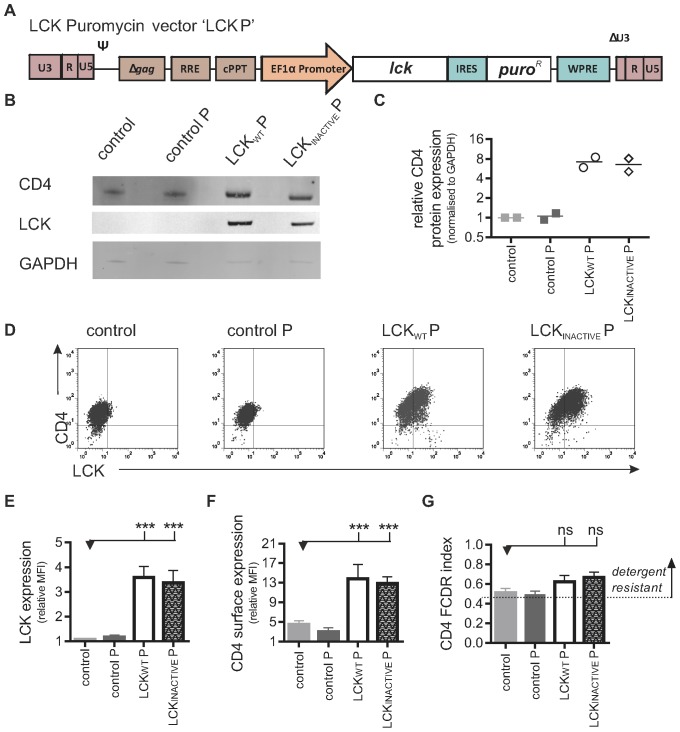
Higher levels of CD4 are found in macrophages expressing LCK. A) Schematic diagram of self-inactivating lentiviral construct used to express wild-type *Lck* and *puro^R^* (LCK_WT_ P). A similar construct was used to express a kinase inactive form of *Lck* and *puro^R^* (LCK_INACTIVE_ P) or to express *puro^R^* only (‘control P’). B) Detection of protein expression of LCK and CD4. Control and transgenic PSC-macrophages lysates were analysed by western blotting using anti-LCK and anti-CD4 antibodies. The loading control GAPDH was detected using anti-GAPDH antibody. C) Protein levels were measured with Odyssey software (Li-COR) and CD4 expression was normalised to GAPDH expression. Symbols represent normalised CD4 expression, relative to the PSC-macrophages control group, of two independent experiments. D) Detection of surface CD4 and total LCK expression. Representative two-colour immunofluorescence (dot plot) analysis is shown. Gates were determined by using the two relevant isotype control antibodies. Quantification of total LCK expression (E) and surface CD4 expression (F), expressed as the ratio of the geometric mean fluorescence intensity (MFI) over the isotype control ±SEM of independent experiments (n = 7). G) Detergent resistance of CD4, expressed as mean Flow Cytometric Detergent Resistance (FCDR) index of CD4 (n = 4) in PSC-macrophages, calculated as described in materials and methods.

The role of the sub-cellular CD4 distribution in HIV-1 entry into macrophages was first investigated by expressing LCK heterologously in macrophages. Previously, it has been shown that the introduction of LCK into myeloid cell lines, or the co-introduction of CD4 and LCK in non-lymphoid cell lines which normally do not express either protein, results in association between LCK and CD4, leading to inhibition of CD4 endocytosis, reduction of the CD4 internalization rate, high levels of surface CD4, and an increase in HIV-1 infection [13], [32]–[36]. As CD4 density on macrophages is highly correlated with infectivity, one would expect that, as has been found in the above studies on cell lines, increasing levels of CD4 would result in increased HIV-1 infection in authentic macrophages [37], [38]. However, myeloid cell lines may not accurately replicate the HIV-1 entry pathway of primary macrophages, so using the lentiviral expression system, exogenous LCK was expressed for the first time in a physiologically relevant human macrophage. LCK_WT_ (LCK_WT_ P) or a kinase-inactive version of the protein (LCK_INACTIVE_ P) were expressed, along with the gene for puromycin resistance, under the control of the EF1α promoter (Figure 1A). Earlier work had shown that transgenes can be successfully and stably expressed in PSC-macrophages using this design (unpublished data). The control vector (henceforth called ‘control P’) lacks the LCK_WT_ or LCK_INACTIVE_ cistron but expresses the puromycin resistance gene. Untransduced cells, differentiated in parallel without puromycin, served as an additional control. Western blot and flow cytometry analysis confirmed that LCK could be expressed in PSC-macrophages (Figure 1B–F), resulting in higher levels of CD4 expression compared to the control macrophages, consistent with previous reports using immortalized myeloid cell lines expressing LCK [32].

CD4 has previously been shown to localize to DRMs in macrophages, and since LCK associates with rafts due to myristoylation at glycine at position 2 at the N-terminus and palmitoylation of at least one of the two cysteine residues at position 3 and 5, expression of LCK in macrophages would be expected to further stabilise CD4 to DRMs [18], [39]–[42]. To confirm the detergent resistance of CD4 in control and LCK+ macrophages, an established flow cytometric assay of differential detergent resistance (FCDR) was performed (developed by *Gombos et al.* [43]). This assay has been validated in a range of cell types, including primary monocytes, T cells, B-cells and various cell lines [44]–[49]. Although the FCDR is highly reproducible, the assay has never been used with macrophages. Therefore, the method was first validated, as shown in Figure S2. Using this assay, the extent of detergent resistance (FCDR index) can be estimated. A value close to 0 is obtained if a surface protein is highly soluble after detergent treatment (i.e. does not localize to DRM) and a value close to 1 represents an insoluble surface protein after detergent treatment (i.e. localizes to DRM). The FCDR index of CD4 (Figure 1G) in Control (mean ±SEM: 0.5±0.04, n = 4), Control P (mean ±SEM: 0.5±0.04, n = 4), LCK_WT_ (mean ±SEM: 0.6±0.06, n = 4) and LCK_INACTIVE_ (mean ±SEM: 0.7±0.06, n = 4) macrophages was higher than the cut-off of 0.45 and therefore considered to be detergent resistant and raft-associated [43], [47]. Expression of LCK did not significantly affect the association of CD4 with DRM as measured by this assay.

### CD4 is limiting for HIV-1 fusion in macrophages

**Figure 2:**
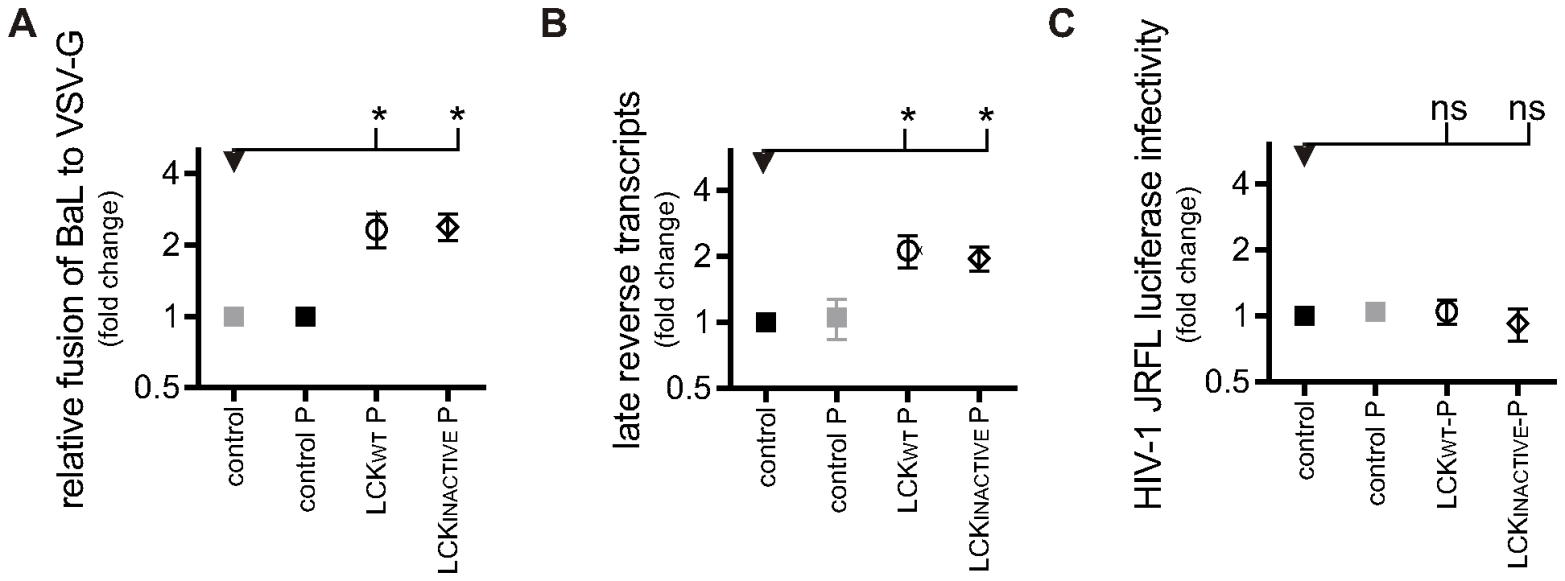
Increased HIV-1 entry in LCK^+^ macrophages results in increased reverse transcription, but not successful infection. A) HIV-1 viral fusion in PSC-macrophages. The percentage of fusion of HIV-1 virions was normalised to the percentage fusion of VSV-G pseudotyped virions. Symbols represent the normalised mean fusion, relative to the PSC-macrophages control group ±SEM of independent experiments. Control-, LCK_WT_ P and LCK_INACTIVE_ P (n = 3) and control P PSC-macrophages (n = 1). B) Reverse transcription of HIV-1 in PSC-macrophages. Late (pol) products were detected by qPCR after 30 h of infection. Symbols represent the relative mean number of copies of HIV-1 DNA±SEM of independent experiments (n = 3), normalised to the number of cells using a β-actin control. C) HIV-1 productive infection in PSC-macrophages. Infection was measured by detecting luciferase activity in PSC-macrophages three days after transduction with NL4.3.Luc.R-E- virus pseudotyped with HIV-1 or VSV-G envelope. HIV-1 NL4.3.Luc.R-E- transduced cells were normalised to VSV-G NL4.3.Luc.R-E- transduced cells. Symbols represent the mean luciferase detection relative to the PSC-macrophages control group ±SEM of independent experiments (n = 3).

Having shown that LCK expression in macrophages leads to elevated levels of CD4 at the surface, the impact this has on the different stages of HIV-1 infection was evaluated using a panel of assays as readouts for sequential stages of HIV-1 infection. The first assay, as described by *Cavrois et al.*, measures the fusion of HIV-1 envelope with a cell-limiting membrane [50]. As an internal control, the percentage of HIV-1-infected macrophages in which fusion had occurred was normalised to the equivalent percentage in VSV-G infected macrophages, as VSV-G-pseudotyped virions would be expected to enter all cells with equal efficiency, independent of CD4 expression levels [51]. Using this assay, fusion was significantly greater in LCK_WT_ P (mean ±SEM: 2.3±0.38; P≤0.05, n = 3) and LCK_INACTIVE_ P (mean ±SEM: 2.4±0.31; P≤0.05. n = 3) expressing macrophages, compared to control macrophages, indicating that tethering CD4 to the cell surface leads to increased HIV-1 fusion in macrophages (Figure 2A).

### Increased HIV-1 entry in LCK^+^ macrophages results in increased reverse transcription, but not increased successful infection

Once virions gain access to the cell cytoplasm an essential step in the HIV-1 life cycle is reverse transcription: the conversion of viral RNA into DNA. Compared to control macrophages, 2.1X and 1.9X more copies of late reverse transcript per cell were detected by qPCR in macrophages expressing LCK_WT_ P and LCK_INACTIVE_ P respectively (Figure 2B). This shows that, in addition to increased fusion, tethering CD4 to the cell surface leads to significantly (P≤0.05) increased HIV-1 reverse transcription. The first two assays measured steps that are necessary but not sufficient for productive entry. Only once the HIV-1 DNA copy is integrated into the host's genome, can it be expressed, and new viral particles produced. The third assay uses a replication-defective pseudotyped HIV-1 that has the gene for luciferase inserted in place of the *nef* gene, and can therefore function as a reporter gene for productive infection (Figure 2C). There was no significant difference in detection of luciferase protein produced in lysates from macrophages expressing LCK_WT_ P or LCK_INACTIVE_ P compared to control macrophages that had been infected with HIV-1 (Figure 2C; mean ±SEM LCK_WT_ P: 1.0±0.14, n = 3; mean ±SEM LCK_INACTIVE_ P: 0.9±0.15, n = 3). In this set of experiments, as with all previous, there was no significant difference between LCK_WT_ P and LCK_INACTIVE_ P, providing assurance that the results observed are not due to signalling pathways activated by introduction of kinase-active LCK. Overall, this set of results suggest that when CD4 is tethered to the cell surface in macrophages, HIV-1 fusion and reverse transcription are enhanced, but this does not result in an increase in viruses following a productive entry route. This implies that diverting CD4 away from its normal recycling route causes HIV-1 to follow an unproductive pathway, most likely involving fusion proximal to/at the surface membrane. If productive HIV infection in macrophages occurred by surface fusion, enhancing plasma membrane fusion by tethering CD4 to the cell surface through LCK expression should have resulted in increased infection efficiency during all stages of the HIV-1 entry pathway. As no increase in productive infection was observed, these results therefore support the model of a non-surface fusion entry route in macrophages as the most efficient productive entry pathway.

### Generation of HIV-1 resistant macrophages using CD4-knock-down

**Figure 3:**
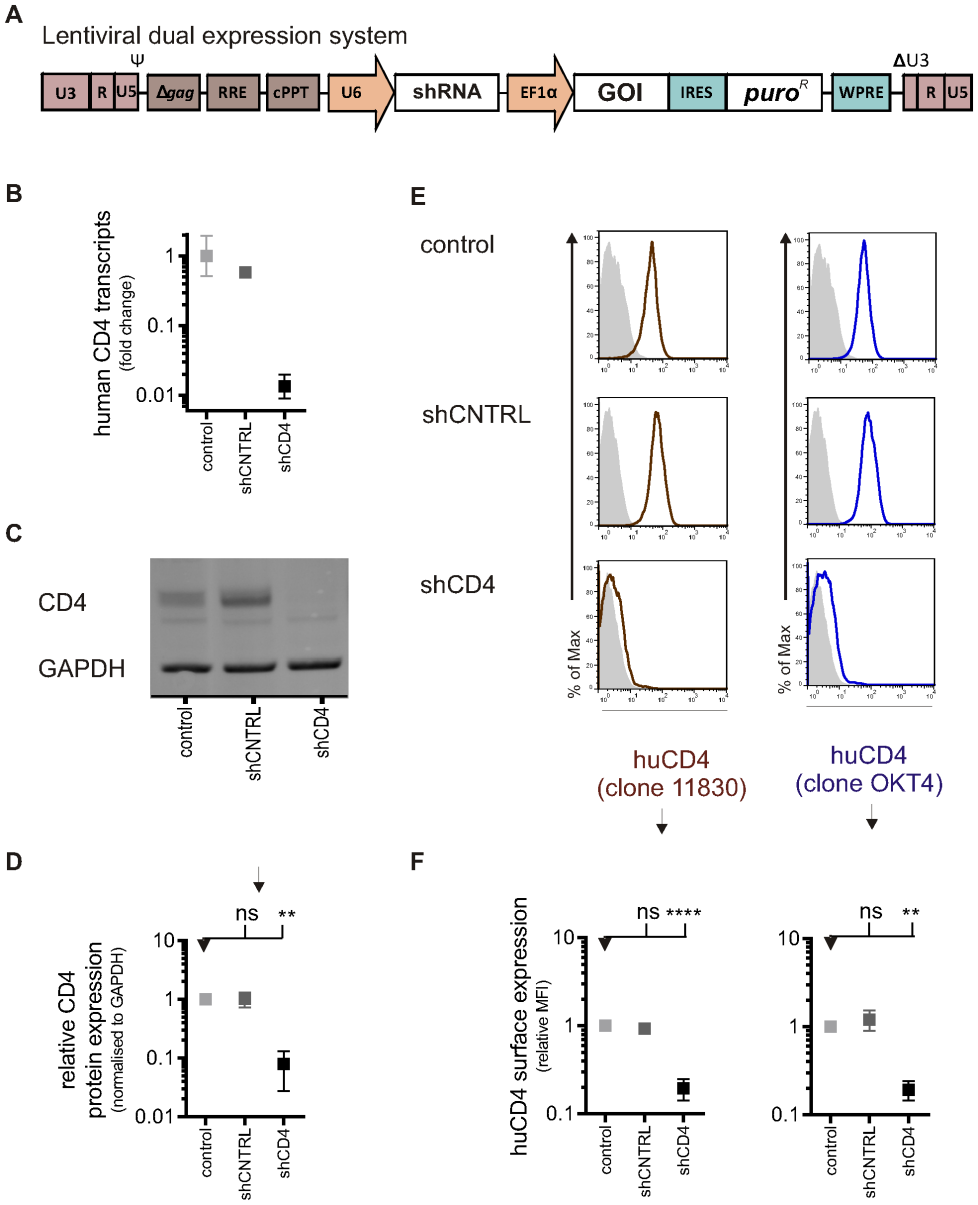
CD4 knock-down in genetically modified stem cell-derived macrophages. A) Schematic diagrams of self-inactivating lentiviral constructs used to express a shRNA (non-specific short-hairpin control (shCNTRL) or a short hairpin targeting CD4 (shCD4)) and puromycin resistance gene (*puro^R^*). The Gene Of Interest (GOI) linked to expression of Puromycin^R^ can easily be cloned downstream from the EF1α promoter. B) Detection of CD4 transcripts. RNA was isolated from control and transgenic PSC-macrophages and analysed by RT-qPCR. Symbols represent the relative mean number of copies of CD4 mRNA±SEM of technical replicates (n = 3) using pooled RNA from three independent experiments. C) Detection of protein expression of CD4. Control and transgenic PSC-macrophages lysates were analysed by western blotting using anti-CD4 antibodies. GAPDH, a loading control, was detected using anti-GAPDH antibody. Representative blot is shown.D) Protein levels were measured with Odyssey software (Li-COR) and CD4 expression was normalised to GAPDH expression. Symbols represent mean normalised CD4 expression relative to the PSC-macrophages control group, ±SEM (n = 3 independent experiments). E) Detection of surface protein expression of CD4. Control and transgenic PSC-macrophages were tested for surface CD4 expression by flow cytometry using two different clones of anti-CD4 antibodies. Representative histograms showing CD4 surface staining with mAb clone 11830 (red/brown line, left panel) and with mAb clone OKT4 (blue line, right panel), both compared to isotype control (shaded gray). The expected phenotype (presence or absence of endogenous human CD4) in cells expressing this lentiviral vector is indicated by the symbols. F) Quantification of CD4 expression with mAb clone 11830 (left) and with mAb clone OKT4 (right) relative to the PSC-macrophages control group. The bars reflect the ratio of the geometric mean fluorescence intensity (MFI) over the isotype control ±SEM of independent experiments (n = 4).

A suitable system that allows effective knock-down of gene expression and allows concomitant transgene expression in PSC-derived macrophages is not trivial, as the vector must be delivered to the PSC and expression must be maintained at acceptable levels post-differentiation in the derived macrophages [22]. In this study, an effective lentiviral dual-expression system was created: the U6 promoter drives the expression of an shRNA, and the EF1α promoter drives the expression of a GOI (Figure 3A). The effectiveness was demonstrated here for the first time by knocking down the HIV-1 receptor CD4 in macrophages and measuring the effect on HIV-1 infection. CD4 is the main HIV-1 receptor in both macrophages and T cells [6], [52]. In macrophages this is supported by plenty of evidence using recombinant soluble CD4 and anti-CD4 monoclonal antibodies, which block infection and ablate infection-related cytopathic effects by several macrophage-tropic HIV isolates, but this has not been tested genetically [1]–[5]. Moreover, unlike in T cells, inhibition of HIV-1 infection in macrophages has not been demonstrated by the use of lentiviral expression of shRNAs targeting CD4 (shCD4) and as macrophage-tropic HIV-1 strains are uniquely able to utilise the low levels of CD4 present in macrophages, it is possible that even effective shRNA knock-down would be insufficient to prevent infection [36], [53]–[55]. PSC were transduced with lentiviral vectors encoding a control hairpin (shCNTRL) or a hairpin targeting CD4 (shCD4), selected with puromycin and differentiated into PSC-macrophages. RT-qPCR analysis showed that CD4 transcripts were reduced by a factor of 100 in shCD4-macrophages compared to control (Figure 3B). CD4 protein expression was reduced by 92% in shCD4-macrophages (mean ±SEM: 0.08±0.05, n = 3; P≤0.01) compared to the control macrophages, whereas shCNTRL-macrophages did not have significantly different CD4 expression levels (mean ±SEM: 1.0±0.29, n = 3; ns) compared to control macrophages (Figure 3C and 3D). The relative surface CD4 expression level (Figure 3E), measured using mAb clone 11830, which recognises domain 1 of CD4, was reduced to background levels on shCD4-macrophages (mean ±SEM: 0.2±0.05, n = 4; P≤0.0001) compared to control macrophages (mean set to 1), but unaffected on shCNTRL-macrophages (mean ±SEM: 1.2±0.31, n = 4; ns) (Figure 3F, left panel). Similar results were obtained using mAb clone OKT4, which detects domain 3 of CD4 (shCD4 mean ±SEM: 0.2±0.05, n = 4; P≤0.01; shCNTRL mean ±SEM: 0.9±0.07, n = 4; ns) (Figure 3F, right panel). Together these results validate the lentiviral expression system for delivery of constructs to PSC and effective knock-down in downstream PSC-macrophages.

**Figure 4:**
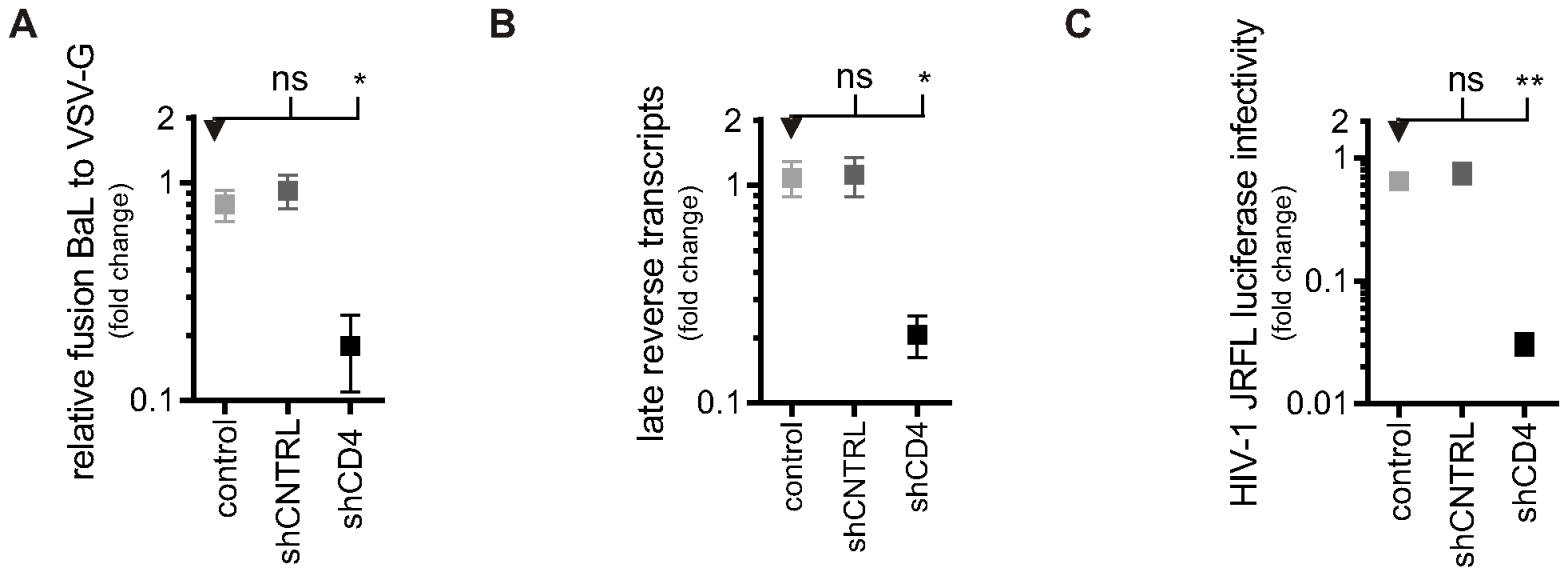
HIV-1 infection is reduced in CD4-knock-down macrophages. A) HIV-1 viral fusion in PSC-macrophages. The percentage of fusion of HIV-1 virions was normalised to the percentage fusion of VSV-G-pseudotyped virions. Symbols represent the normalised mean fusion relative to the PSC-macrophages CD4_WT_ group (shown in Figure 7) of four independent experiments ±SEM (n = 4). B) Reverse transcription of HIV-1 in PSC-macrophages. Late (pol) products were detected by qPCR after 30 h of infection. Symbols represent the relative mean number of copies of HIV-1 DNA±SEM of independent experiments (n = 4), normalised to the number of cells using a β-actin control. C) HIV-1 productive infection in PSC-macrophages Infection was measured by detecting luciferase activity in PSC-macrophages three days after transduction with NL4.3.Luc.R-E- virus pseudotyped with HIV-1 or VSV-G envelope. HIV-1 NL4.3.Luc.R-E- transduced cells were normalised to VSV-G NL4.3.Luc.R-E- transduced cells. Symbols represent the mean luciferase detection relative to the PSC-macrophages CD4_WT_ group (shown in Figure 7) ±SEM of independent experiments (n = 4).

Next, HIV-1 infection in shCD4-macrophages was assessed. HIV-1 fusion was significantly lower in shCD4-macrophages (mean ±SEM: 0.2±0.07, n = 4; P≤0.01), compared to control macrophages (mean ±SEM: 0.8±0.11, n = 4), but was not significantly different in shCNTRL-macrophages (mean ±SEM: 0.9±0.16, n = 4; ns) (Figure 4A).

Next, reverse transcription was assessed in shCD4-macrophages (Figure 4B). Significantly fewer copies of late reverse transcripts per cell were detected in shCD4-macrophages (mean ±SEM: 0.2±0.04, n = 4; P≤0.05) compared to control macrophages (mean ±SEM: 1.1±0.19, n = 4), while similar copy numbers were detected in shCNTRL-macrophages (mean ±SEM: 1.1±0.23, n = 4; ns). Finally, productive infection was measured using the reporter assay for HIV-1 integrants. (Figure 4C). Compared to control macrophages (mean ±SEM: 0.6±0.05), detection of luciferase protein produced in lysates from shCD4-macrophages was reduced 20.6-fold (mean ±SEM: 0.03±0.01, n = 4; P≤0.01), whilst it was unchanged in shCNTRL-macrophages (mean ±SEM: 0.76±0.15, n = 4; ns).

Together, these assays demonstrate, as expected, that when CD4 is knocked down in shCD4-macrophages HIV-1 fusion, reverse transcription, as well as productive infection are all reduced. These results demonstrate the principle of using shRNA to study HIV-1 infection in PSC-macrophages and provide a system to investigate CD4 function using knock-in mutants on the null background.

### Alteration of CD4 association with detergent resistant membranes affects HIV-1 infection of macrophages

**Figure 5:**
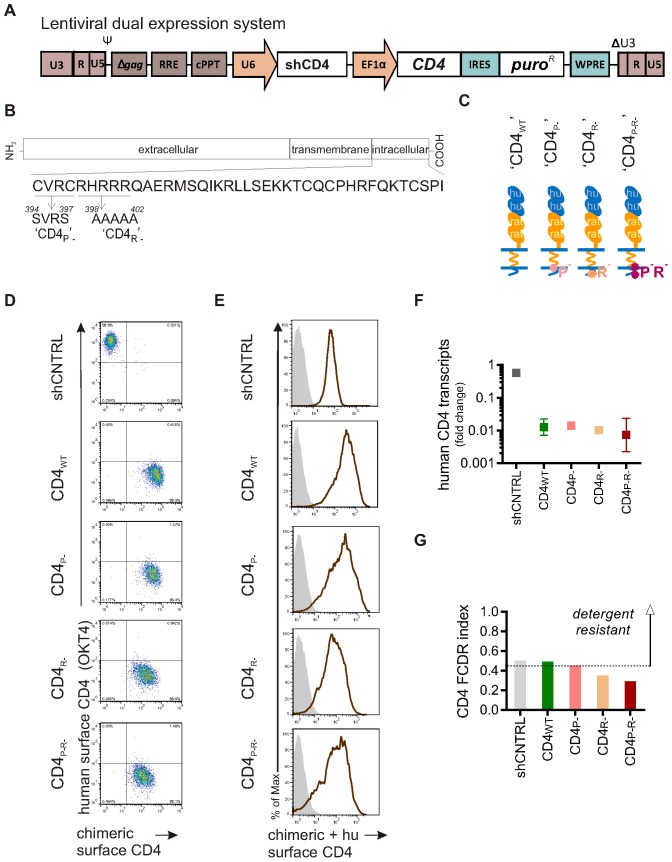
CD4 localization to detergent resistant membranes is reduced in CD4- mutants compared to wild-type CD4 in macrophages. A) Schematic diagram of self-inactivating dual-expression lentiviral vector used to knock-down endogenous CD4 and express *puro^R^* and chimeric CD4. B) Amino acid sequence of cytoplasmic tail of CD4 showing location of regions involved with DRM localization and the mutants inserted to produce CD4_P-_, CD4_R-_ and CD4_P-R-_. C) Symbols used to highlight the structure of each mutant chimeric CD4. Chimeric CD4 contains domain 1+2 and the cytoplasmic tail of human CD4 (depicted in blue), but domain 3+4 and transmembrane domain of rat CD4 (depicted in orange). D) Validation of the design of the lentiviral constructs expressing the chimeric CD4, TZM-bl cells were transduced with lentiviral vectors encoding shCD4 and wild-type (WT) or mutant versions of chimeric CD4 (P-, R-, or P-R-) or with a lentiviral vector expressing a control shRNA only (shCNTRL). Two-colour immunofluorescence (dot plot), showing the mean fluorescence intensities of mAb clones OKT4 and OX68, plotted on the Y-axis and X-axis, respectively. Gates were determined by using the two relevant isotype control antibodies. anti-CD4 mAb clone OKT4 recognises endogenous CD4 (domain 3 of human CD4) and anti-CD4 mAb clone OX68 recognises chimeric CD4 (domain 3+4 of rat CD4). E) Detection of surface expression of CD4 in PSC-macrophages. Histogram showing CD4 surface staining of PSC-macrophages with mAB clone 11830 (red/brown line) compared to the matched isotype control (shaded grey). anti-CD4 mAb clone 11830 recognises both endogenous human CD4 and chimeric CD4 (domain 1). F) Detection of CD4 transcripts. RNA was isolated from PSC-macrophages and analysed by RT-qPCR using specific primers. Symbols represent the relative mean number of copies of human endogenous CD4 mRNA relative to the control group (shown in Figure 4A) ±SEM of technical replicates (n = 3) using pooled RNA from three independent experiments. G) Detergent resistance of CD4. Bars reflect the mean Flow Cytometric Detergent Resistance (FCDR) index of CD4 in PSC-macrophages, calculated as described in materials and methods.

In order to investigate the physiological relevance of CD4 association to DRM for HIV-1 infection, we used our dual-expression system (Figure 5A) to knock-down CD4, and also simultaneously expressed CD4 mutants known to have reduced association with DRM. The mutants were based on two regions in the cytoplasmic tail of CD4 that have been shown to be important for the DRM localization of CD4 (Figure 5B). The first region is composed of two cysteines at position 394 and 397 that are palmitoylated in wild-type CD4, but not in CD4 in which these cysteines are substituted with serines [56], [57]. This mutant, referred to as ‘CD4_P-_’ in this study, has previously been shown to result in reduced DRM-localization in a range of cell types, including primary T cells and cell lines, as determined biochemically using Triton X-100 solubilisation and by confocal microscopy [56], [58]–[60]. Nonetheless, palmitoylation may not be absolutely necessary or sufficient for CD4 DRM localization, considering that *Popik et al.* reported that irrespective of CD4 palmitoylation a second region in the cytoplasmic tail of CD4, a short sequence of positively charged arginine and histidine residues (RHRRR), may control CD4 partitioning to DRM [57]. Substitution of these five residues with alanine residues, resulting in a mutant referred to as ‘CD4_R-_’ in this study, excludes CD4 from DRM, as determined by Triton X-100 solubilisation and by confocal microscopy using a B-cell line [57].

To be able to differentiate between endogenous CD4 and exogenous CD4, and for the exogenous CD4 to be resistant to the shCD4, a human-rat chimeric CD4 was designed. Residues in domain 1 of human CD4 are required for the interaction with gp120 and HIV-1 infection, so domain 1 and 2 of the chimera were derived from the human gene [61]–[64]. Domains 3+4 and the transmembrane of rat CD4 were used in the chimera in order that the mutant CD4 could be easily recognized and distinguished from endogenous CD4 with a specific anti-CD4 rat antibody (clone OX68) [65]. The cytoplasmic tail of human CD4 was used to specifically manipulate the sub-cellular distribution of CD4 by creating the mutant versions of chimeric CD4 (CD4_P-_ and CD4_R-_) or a double mutant (CD4_P-R-_). The constructs are schematically illustrated Figure 5C.

To design and expression of chimeric CD4 using the lentiviral dual knock-down knock-in expression system was validated in TZM-bl cells, a HeLa derivative that expresses high levels of CD4 and CCR5, using two-colour immunofluorescence flow cytometry [66], [67]. CD4 levels in cells transduced with lentiviruses encoding shCD4 and chimeric CD4 (CD4_WT_, CD4_P-_, CD4_R-_, or CD4_P-R-_) were reduced by 98%–99%, while a high endogenous CD4 level was retained in shCNTRL cells (Figure 5D). This shows that the hairpin targeting CD4, expressed under the control of the U6 promoter, is not affected by the expression of chimeric CD4 on the same lentiviral vector, and can still effectively knock-down endogenous CD4 to background levels. Also, the CD4 mutants were all expressed at similar levels (Figure S3).

Next, macrophages were derived from PSC transduced with the dual-expression vectors CD4_WT_, CD4_P-_, CD4_R-_, or CD4_P-R_. qPCR and flow cytometry analysis confirmed CD4 knock-down and expression of the chimeras in these macrophages (Figure S3 and Figure S4). Expression levels of chimeric CD4 were at least as high as endogenous CD4 levels on control cells, as shown by flow cytometry staining with mAb clone 11830, recognising Domain 1+2 of CD4 (Figure 5E; and Figure 3E) and as shown by RT-qPCR analysis (Figure 5F). This confirms that the structural similarities between human and rat CD4 allow the design of a chimeric CD4 version that can be successfully expressed at the cell surface.

The FCDR assay (Figure 5G) for DRM association indicated that both endogenous wild-type CD4 (shCNTRL FCDR index: 0.50) and chimeric wild-type CD4 (CD4_WT_ FCDR index: 0.49) were relatively resistant to detergent treatment in macrophages, whereas the raft mutant (CD4_R-_ FCDR index: 0.35) and double mutant (CD4_P-R-_ FCDR index: 0.29) were more sensitive to detergent treatment in macrophages. The palmitoylation mutant (CD4_P-_ FCDR index: 0.45) had marginally reduced sensitivity to detergent compared to wild-type CD4.

### CD4 localization to detergent-resistant membranes is required for productive HIV-1 infection in macrophages

**Figure 6:**
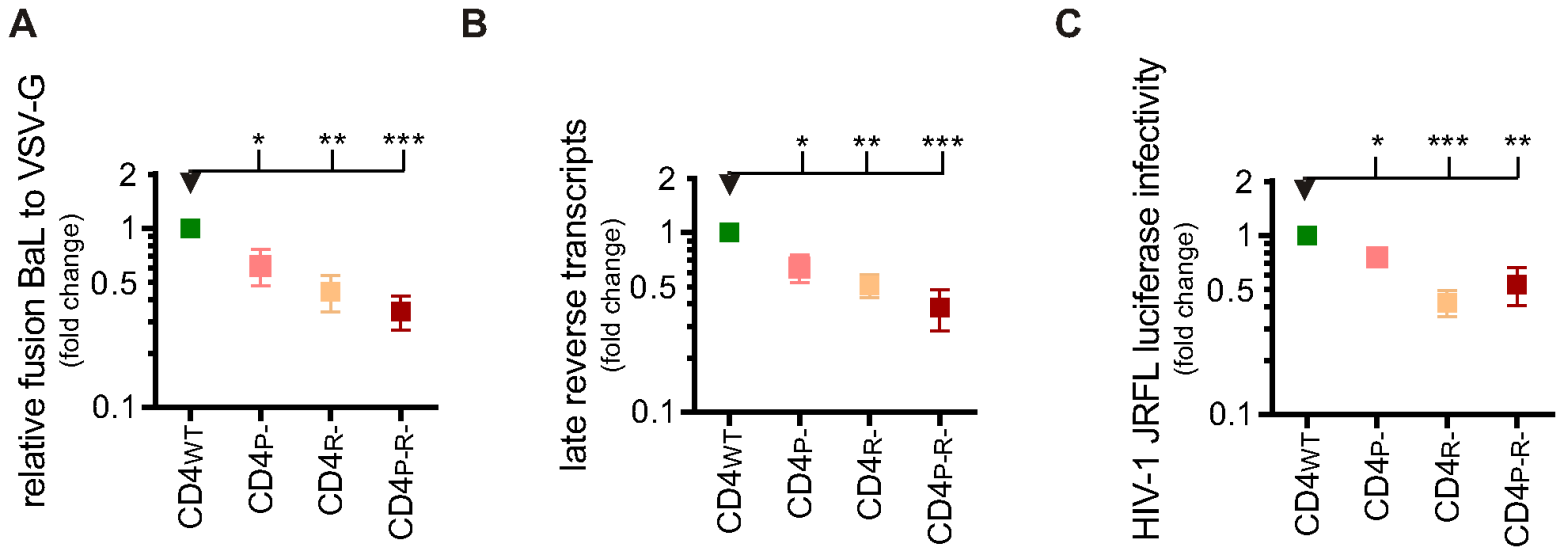
HIV-1 infection is reduced when CD4 is excluded from DRM in macrophages. A) HIV-1 viral fusion in PSC-macrophages. The percentage of fusion of HIV-1 virions was normalised to the percentage fusion of VSV-G pseudotyped virions. Symbols represent the normalised mean fusion relative to the PSC-macrophages CD4_WT_ group of four independent experiments ±SEM (n = 4). B) Reverse transcription of HIV-1 in PSC-macrophages. Late (pol) products were detected using qPCR after 30 h of infection. Symbols represent the relative mean number of copies of HIV-1 DNA of four independent experiments ±SEM (n = 4), normalised to the number of cells using a β-actin control. C) HIV-1 productive infection in PSC-macrophages. Infection was measured by detecting luciferase activity in PSC-macrophages three days after transduction with NL4.3.Luc.R-E- virus pseudotyped with HIV-1 or VSV-G envelope. HIV-1 NL4.3.Luc.R-E- transduced cells were normalised to VSV-G NL4.3.Luc.R-E- transduced cells.Symbols represent the mean luciferase detection relative to the PSC-macrophages CD4_WT_ group of six independent experiments, ±SEM (n = 6).

The impact of the CD4_P-_, CD4_R-_ and CD4_P-R-_ mutants on the different stages of HIV-1 infection in macrophages was evaluated and compared to CD4_WT_ macrophages HIV-1 fusion (Figure 6A) in CD4_WT_ macrophages (mean set to 1) was similar to the engineered macrophages expressing endogenous human CD4 (shCNTRL, shown in Figure 4; mean ±SEM: 0.9±0.16, n = 4; ns). This demonstrates that chimeric CD4 can act as a ‘functional’ HIV receptor. Meanwhile, fusion was significantly lower in all three CD4 mutants: CD4_P-_ (mean ±SEM: 0.6±0.14, n = 4; P≤0.05), CD4_R-_ (mean ±SEM: 0.4±0.10, n = 4; P≤0.01) and CD4_P-R-_ (mean ±SEM: 0.3±0.07, n = 4; P≤0.001). These results indicate that CD4 exclusion from DRM leads to reduced HIV-1 fusion in macrophages and implies that CD4 localization to DRM is required for HIV-1 entry. In addition to reduced HIV-1 fusion, CD4 exclusion from DRM results in reduced HIV-1 reverse transcription (Figure 6B). Compared to CD4_WT_ macrophages (mean set to 1), fewer copies of late reverse transcript per cell were detected in all three CD4 mutants: CD4_P-_ (mean ±SEM: 0.6±0.11, n = 4; P≤0.05), CD4_R-_ (mean ±SEM: 0.5±0.08, n = 4; P≤0.01) and CD4_P-R-_ (mean ±SEM: 0.4±0.10, n = 4; P≤0.001) (Figure 6B). Finally, the reporter assay from HIV-1 integrants provided further evidence that CD4 localization to lipid rafts is required for productive infection (Figure 6C). Compared to CD4_WT_ macrophages (mean set to 1), detection of luciferase protein produced in lysates from macrophages expressing the CD4 mutants was significantly reduced (CD4_P-_ mean ±SEM: 0.8±0.08, n = 6; P≤0.05; CD4_R-_ mean ±SEM: 0.4±0.07, n = 6; P≤0.001; CD4_P-R-_ mean ±SEM: 0.5±0.13, n = 6; P≤0.01). Overall, this set of three assays provides consistent evidence that redistribution of CD4 to detergent-susceptible regions of the membrane results in a reduction of productive entry of HIV-1 in macrophages.

## Discussion

**Figure 7:**
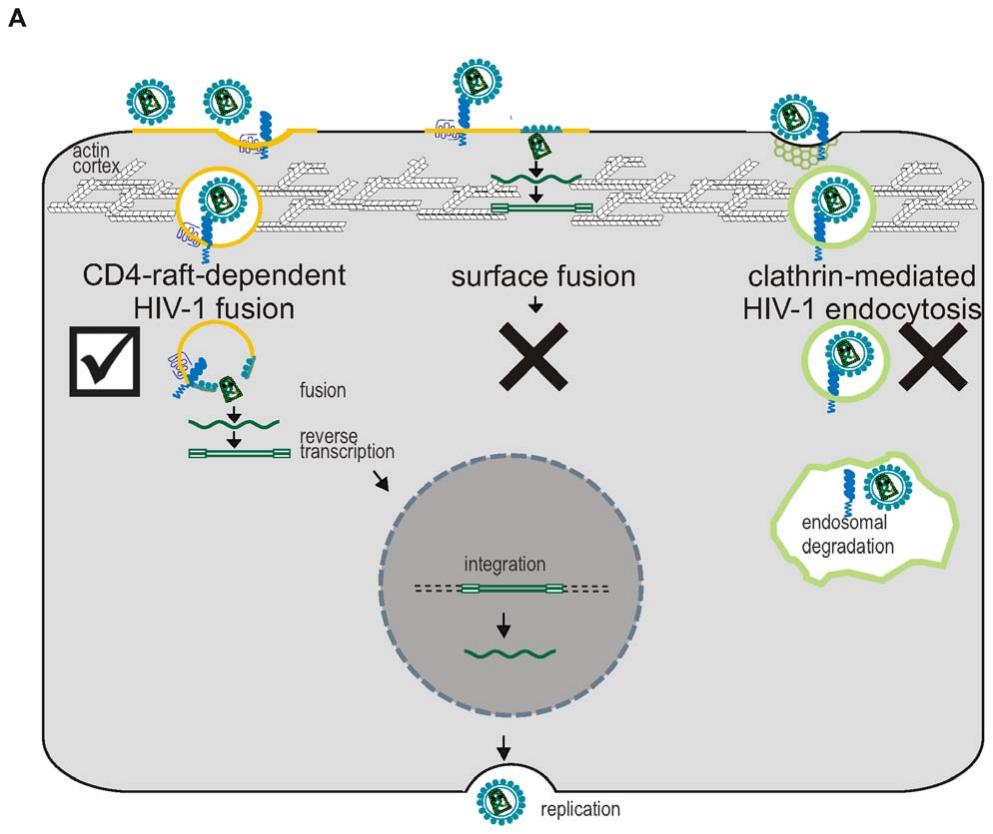
Proposed model of HIV-1 entry in macrophages. A) Virions can be taken up non-specifically via endocytic routes (left and right), or via fusion with the cell surface (middle). Plasma membrane fusion is likely a dead-end pathway, as few reverse transcripts reach the nucleus, presumably due to the barrier formed by the cortical actin. Macrophages have low levels of surface CD4, thereby favouring virion uptake through endocytosis over plasma membrane fusion. Endocytic routes naturally overcome the actin barrier and facilitate productive infection, providing that the virion engages with CD4/CCR5 within the endosome and fuse from within this compartment prior to degradation. Virions are more likely to fuse from a raft-dependent uptake pathway (left) and escape endosomal degradation or recycling (not depicted in figure) compared to other endocytic routes, such as clathrin-mediated endocytosis (right), as the co-receptor CCR5 associates with lipid raft microdomains in macrophages.

In this work we have provided data supporting the idea that HIV-1 entry into macrophages results from fusion after uptake of virions by a CD4-raft-dependent pathway. The data has been acquired using a novel approach to the genetic modification of macrophages, and as such it provides a valuable alternative line of evidence to previous drug-based studies, with significant advantages such as reduced off-target effects. One proposed model for HIV-1 entry, based on this study and others [15], [18], [19], [68]–[70], is illustrated in Figure 7, in which fusion at the plasma membrane does not result in productive infection, presumably due to the barrier formed by the actin cortex and the presence of intrinsic antiviral factors [71]. This is supported by our data, as tethering CD4 to the cell surface, using LCK, increased HIV-1 fusion but did not increase productive infection. The model proposes instead, that an endocytic HIV-1 entry route in macrophages would be more productive, which would allow HIV-1 to overcome the actin barrier and cover some of the distance towards the nucleus before fusing with the host cell membrane [68], [69].

Fusion would need to occur before degradation of the endosomal contents, and as CD4 and CCR5 localize preferentially to raft micro-domains in macrophages [18], raft-dependent endocytic uptake of HIV-1 is the most likely route to enable efficient fusion of HIV-1 after endocytosis, and therefore escape into the cytosol before degradation. The data presented here support this model, as DRM mutants of CD4 led to decreased productive HIV-1 infection. The CD4-raft dependent pathway that can lead to productive infection in macrophages has been characterized in more detail by *Carter et al.* [18], [19], termed the “Pathway of HIV Endocytic Entry in Macrophages” (PHEEM). In addition to the requirement for CD4 localization to lipid rafts, PHEEM is dependent on intact lipid rafts and involves Na+/H+ exchange, actin rearrangement, dynamin, Rho family GTPases, and Pak1, but not PI-3 kinase or myosin II, and it requires CCR5 engagement to induce HIV-1 internalization [16], [19]. Fusion from clathrin-coated vesicles is presumably less successful due to the relative scarcity of CCR5 in these vesicles [18], and the relatively higher likelihood of degradation before fusion can occur, or return to the plasma membrane through recycling endosomes [15], [16], [72]. With the proposed model of HIV-1 entry, the ability of HIV-1 to establish successful infection associated with a certain entry pathway can be viewed as a spectrum, which depends on the balance between virus fusion kinetics (influenced by (co-)receptor expression levels and raft localization) and endolysosomal degradation rates, as well as the penetration of the actin barrier and the distance to the nucleus [19], [70]. Low levels of surface CD4, as normally found on the surface of macrophages, would result in low fusion kinetics. This would lead to the accumulation of unfused virions at the cell surface and increased endocytic uptake. Therefore, it has been suggested that low surface CD4 levels favour virion uptake through endocytosis over unproductive plasma membrane fusion [73]. Actin has a paradoxical role in this model, as it facilitates the formation of HIV-1 endocytic uptake vesicles, but also forms a barrier to virions that have fused at the cell surface [71]. This model is in agreement with HIV-1 entry models proposed by *Permanyer et al.* and *Gobeil et al.* and emphasises the diverse routes by which HIV-1 can gain entry to the cell, and how the productive route may be cell type specific [70], [73].

Detergent resistance is widely used operationally to define lipid rafts, although the definition of “lipid rafts” requires additional evidence, such as evaluation of size, mobility, and identification of presence of raft markers, such as sphingolipids, cholesterol and raft-associated proteins [74]–[78]. The raft localization region of CD4 (^398^RHRRR^402^) has been suggested to be the dominant signal for raft targeting of CD4 in a B-cell line, but has not been studied further in other cell types [57]. Here, macrophages expressing CD4_R-_ had a lower FCDR index compared to CD4_P-_, which may suggest that as with B-cells, ^398^RHRRR^402^ is the dominant raft signal in macrophages. Reducing the proportion of CD4 in DRM in macrophages using CD4_P-_, CD4_R-_ or CD4_P-R-_ mutants, in all cases led to a decrease in infection as observed using three different HIV-1 assays. This effect was more pronounced in the DRM excluded CD4_R-_ compared to the partially DRM excluded mutant CD4_P-_. This provides direct evidence for the requirement for CD4 raft localization in HIV infection of macrophages. Lipid rafts are rich in components that are recognized by HIV-1. The envelope protein subunit gp41 is palmitoylated on two cysteine residues, modifications that promote lipid raft association [79], [80]. The envelope protein subunit gp120 contains a common sphingolipid-binding motif through which it interacts with various sphingolipids enriched in lipid rafts, including ceramides, gangliosides and sphingomyelin [79]–[86]. These glycosphingolipids can mediate the clustering of rafts and aid the organisation of the membrane fusion complex containing the HIV-1 envelope, CD4 and CCR5 [87]–[89]. In addition to lipids, HIV-1 gp120 can attach to other components of the cell surface in macrophages, which have been implicated in various raft-dependent endocytic routes, such as syndecans, [90]–[93]. This suggests that HIV-1 specifically targets the lipid raft regions of the macrophage plasma membrane, through multiple interactions, possibly to focus its binding to an area containing both its receptors and co-receptor, and from which endocytosis can occur. However, further studies are needed to confirm the involvement of these additional raft interactions in HIV-1 entry of macrophages.

## Materials and Methods

### Ethics statements for use of stem cell line

The human ES cell line HUES-2 (passages 16–38) was obtained from the HUES Facility, University of Harvard [94]. Ethical approval for work on all hES cell lines was reviewed and approved by the UK Stem Cell Bank Steering Committee (Medical Research Council, London UK, 20.10.2005).

### PSC culture

PSC were cultured at 37°C with 5% CO2 on Mitomycin C-inactivated mouse embryo fibroblasts (MEFs) in PSC media. PSC media consisted of knock-out DMEM (Invitrogen), 20% knock-out-Serum Replacement (Invitrogen), 2 mM Glutamax-I (Gibco), 100 U/mL penicillin (Invitrogen), 100 µg/mL streptomycin (Invitrogen), 1% non-essential amino acids (Invitrogen), 0.055 mM 2-mercaptoethanol (R&D), 10 ng/mL bFGF (R&D). Passage number was kept within as narrow window as possible to reduce the variability between cultures and the likelihood of karyotypical change.

### Genetic Modification of PSC

For constitutive expression of a transgene, the coding sequence for gene of interest were inserted into the self-inactivating 2^nd^ generation lentiviral vector backbone plasmid pEF1-GOI-IRES-Puro^R^ (based on [95], [96]). The inserted gene of interest (GOI) was, therefore, under the control of the constitutively active Elongation Factor 1α (EF1α) promotor, and was transcribed on a bicistronic transcript together with the Internal Ribosome Entry Sequence (IRES), followed by the gene for resistance to puromycin. At the ribosome, the two genes on the bicistronic transcript are translated as two separate peptides; however, the above arrangement ensures that cells expressing the first gene can be reliably selected with puromycin. The human Lck inserts originated from plasmids expressing Lck wild type (WT), a kinase inactive Lck (Y394F, TAC→TTC). The original sequences were confirmed. For knock-down, the U6 promoter and a control shRNA (shCNTRL) or shRNA targeting CD4 (shCD4) were cloned from the RNAi-Ready pSIREN vector (631526, Clonetech) into pEF1-GOI-IRES-Puro, upstream of the EF1α promoter, creating pU6-shRNA-EF1-GOI-IRES-Puro^R^, The CD4 target sequences (5′→3′: GAACAAGGAAGTGTCTGTA) and control target sequence (5′→3′: ATCATCCCCGACTCGTTTA) have both previously been described [97], [98]. The chimeric wild-type and mutant CD4 sequences were designed and codon-optimized using DNA2.0 GeneDesigner software and produced by GeneArt (Invitrogen). The Genes Of Interest were provided in an Ampicilin^R^ vector pMA-RQ cloned into pU6-shRNA-EF1-GOI-IRES-Puro^R^. Lentivirus was prepared as described previously [22]. Lentivirus was added to PSC (∼70% confluence) with a MOI of approximately 0.08 in a total volume of 1 mL, supplemented with polybrene (5 µg/mL, Sigma) onto one well of a 6-well plate (i.e. approx. 1 million cells) and centrifuged for 1 h 37°C at 1600 *g*. This MOI and target cell number was chosen to achieve a physiologically relevant gene dose (1–2 copies on average), whilst infecting a large enough pool of cells that the line generated was highly polyclonal, with different integrations in different cells and not likely to represent a skewed, karyotypically abnormal subpopulation of the parent population. PSC were incubated at 37°C for 3 h after which the inoculum was replaced with fresh media. PSC were fed as normal for 2 days, and on day 3 puromycin (5 µg/mL, Sigma) was added.

### Directed differentiation of EBs to produce PSC-MC

PSC were mechanically dissociated and transferred into a well of a 6-well ultra-low adherence plate (Corning) in PSC culture medium and cultured for 4 days in PSC media for spontaneous Embryoid Body formation. For haematopoietic differentiation, EBs were transferred into one well of a six-well tissue culture plate and cultured in X-VIVO™15 (Lonza), supplemented with 100 ng/mL M-CSF (Invitrogen), 25 ng/mL IL-3 (R&D), 2 mM glutamax (Invitrogen), 100 U/mL penicillin and 100 µg/mL streptomycin (Invitrogen), and 0.055 mM β-mercaptoethanol (Invitrogen). non-adherent PSC-MC were harvested weekly from the supernatant of EB cultures.

### Macrophage culture and maturation

Monocytes were plated onto tissue culture-treated plates at a density of 1.5×10^5^ cells/cm^2^. Monocytes were maintained in MDM medium (.375×10^6^/mL), consisting of X-VIVO™15 (Lonza) or of RPMI 1640 (PAA) with 10% FCS (PAA). The medium was supplemented with 100 ng/mL (approx. 1.7×10^4^ units/mL) recombinant human M-CSF (R&D Systems), as well as, 2 mM glutamine (PAA), 100 U/mL penicillin and 100 µg/mL streptomycin (PAA). Monocytes were incubated at 37°C, with 5% CO_2_ and differentiated for 5–7 days prior to use. For activation of macrophages for cytokine profile experiments, IFN-gamma (R&D, 100 U/mL) and LPS (Sigma, 100 ng/mL) were added to the culture medium during the last 16 hours of culture. For MDM analysis, cells were detached using cold PBS containing EDTA (5 mM).

### qPCR of endogenous and chimeric CD4 transcripts and integrants

Macrophages were harvested by scraping and pelleted by centrifugation. Total RNA and DNA was extracted using All-Prep RNA/DNA kit (Qiagen) according to the manufacturer's instructions, with on-column DNase digestion. cDNA was produced from the extracted RNA using the Ambion RETROscript kit (1 µg of RNA per reaction with Oligo-dT primer). The cDNA or DNA was used in a SYBR green real-time qPCR reaction using (Applied Biosystems) with the following primers. Human genomic CD4 forward primer 5′→3′: TGGAGTCGCAAGCTGAACTA [99]. Human genomic CD4 reverse primer 5′→3′: CAGAGTGAGAACCTGTCTTGAAAA [99]. Human CD4 cDNA forward primer 5′→3′: GCTGGAATCCAACATCAAGG [100]. Human CD4 cDNA reverse primer 5′→3′: CTTCTGAAACCGGTGAGGAC [100]. Chimeric CD4 genomic/cDNA forward primer 5′→3′: TTTAGAGAGCCCACCCGGTA. Chimeric CD4 genomic/cDNA reverse primer 5′→3′: GCTGGCTCACACTTAGGGTT. To determine relative expression levels (using the ΔΔC(t) method [101]), β-actin DNA and cDNA was measured in parallel using primers from the β-actin control kit (Eurogentec). Reactions contained 12.5 µL *Power* SYBR® Green qPCR Mastermix (Applied Biosystems), 100 nM forward primer, 100 nM reverse primer and 1 ng DNA or cDNA, in a total reaction volume of 25 µL (volume made up with water). The qPCR programme was as follows: 95°C for 10 min, 40 cycles of 95°C for 15 sec, 57.5°C for 1 min, with a plate read after each cycle, followed by a melting curve stage: 15 sec 95°C and 1 min 60°C and reading the plate every 0.3°C to 95°C, where it was held for 15 sec and performed on an Applied Biosystems StepOne Plus Real Time PCR machine, with StepOne software. StepOne software (Applied Biosystems) was used to determine relative expression levels. Error bars represent the SEM based on the relative quantification (RQ)min/RQmax confidence set at 95%.

### Western blotting

Macrophages were lysed (1% v/v n-Dodecyl beta-D-maltoside, 20 mM Tris/HCl pH 8; 150 mM NaCl, containing and phosphatase inhibitors (Sigma)). Cleared lysate was mixed with 4× NuPAGE LDS (Lithium dodecyl sulphate) sample buffer and 10× NuPAGE sample reducing agent (Invitrogen) and heated for 10 min at 70°C. Lysates were electrophoresed at 180 V at RT through NuPAGE® Bis-Tris Precast Gels (Invitrogen). Protein was transferred onto 0.2 µm PVDF membranes, and membranes were incubated with primary mouse anti-human antibodies to CD4 (clone H-370, Santa Cruz), LCK (clone 3A5, Santa Cruz) and GAPDH (clone 2275-PC-100, R&D Systems). To detect primary antibody binding, membranes were incubated with the appropriate LI-COR secondary antibody (goat anti-mouse IgG IR Dye 800/680 or goat anti-rabbit IgG IR Dye 800/680). Protein was detected using the quantitative western blotting imaging system Odyssey (LI-COR).

### Flow cytometry

For analysis of cell surface molecules, cells were washed and stained in flow cytometry buffer consisting of PBS, human IgG (10 µg/mL Sigma), FCS (1% Hyclone) and sodium azide (0.01%), with an antibody or an isotype-matched control (with same fluorophore, from the same manufacturer) on ice for 30 min. For two-colour staining, two antibodies or two isotype controls (attached to different fluorophores) were added together, except for the LCK and CD4 double staining (which is described below). Antibodies used are huCD4 D1 (mIgG2a/APC, clone 11830, R&D Systems), huCD4 D3 (mIgG2b/APC, clone OKT4, Biolegend), ratCD4 D3 (mIgG2a/FITC, AbD Serotec), huCD14 (mIgG1/APC, MEM-15, Immunotools), huCD45 (mIgG1/APC, MEM-28, Immunotools). After the primary staining, cells were washed three times and if unconjugated antibodies were used, stained with a secondary antibody on ice for 30 min and washed another three times. Cells were fixed with 4% formaldehyde in PBS. Fluorescence was measured using a FACS Calibur (Becton Dickinson), and data was analysed using FlowJo software on marked cell populations on FSC-SSC dot plots. For the two-colour staining with CD4 and LCK, cells were first stained for surface CD4 (mIgG2a/APC, clone 11830, R&D systems) and fixed with 4% formaldehyde in PBS and permeabilised using 0.2% saponin (Sigma) in flow cytometry buffer. Next, cells were stained for total LCK expression (rIgG, clone 73A5, Cell Signalling) and stained with a FITC conjugated secondary antibody (sc-2012, Santa Cruz), as described above, except 0.2% saponin was kept present during the washes and staining incubation.

### Flow Cytometric assay of differential detergent resistance

To determine the detergent resistance of CD4, PSC-macrophages were left unlabelled or were labelled with anti-CD4 (clone 11830), as described above, except cells were left unfixed. Cells were treated for 4 min with PBS or with 0.030% Triton X-100 for 4 min. Cells were labelled according to the flow cytometry protocol as described above. Unlabelled cells were used to measure auto-fluorescence (AF). The detergent, Triton X-100 (Sigma) was diluted using PBS to achieve 0.025%, 0.030%, 0.040% (v/v) solutions and cooled on ice. The labelled or unlabelled control cells were mixed with ice-cold detergent (det) or ice-cold PBS (as a control). Fluorescence was immediately measured in the time-resolved mode for 4 min using a FACS Calibur (Becton Dickinson). Also, the end-point mode was used to obtain the MFI before or 4 min after adding the detergent or PBS. Data was analysed using FlowJo software. In order to calculate the FCDR index, the mean fluorescence intensity (MFI) of the following conditions was obtained: labelled, detergent-treated cells (MFI_det_); unlabelled detergent-treated cells (MFI_AFdet_); labelled, PBS-treated cells (MFI_max_); unlabelled PBS-treated cells (MFI_AF_). The extent of detergent resistance was calculated as follows: FCDR = (MFI_det_−MFI_AFdet_)/(MFI_max_−MFI_AF_).

### HIV-1 virion fusion assay

A replication-defective pseudotyped HIV-1 allows the examination of single-cycle HIV-1 infection. The commonly used replication-defective virus is NL4-3.Luc.R-E- (NIH AIDS Research and Reference Reagent Program, Division of AIDS, NIAID, NIH from Dr. Nathaniel Landau) was used to measure HIV-1 fusion [102], [103]. The NL4-3.Luc.R-E can be pseudotyped with different viral envelopes. VSV-G enveloped viruses use an ubiquitous membrane component (phospholipid) to enter cells, through clathrin-mediated endocytosis and pH dependent fusion [104], [105]. Because entry of VSV-G pseudotyped viruses is independent of CD4 expression levels and distribution, it can be used as a control to normalise to the number of cells present. BaL and VSV-G pseudotyped HIV-1 NL4.3.Luc.R-E containing β-lactamase-Vpr chimera (BlaM-Vpr), capable of single round of replication were prepared as described previously [22], except the following amounts of plasmid were used per well of a 6-well plate: 0.6 µg pVpr-Blam, 1.8 µg pNL4.3 luc and 1.2 µg VSV-G or BaL (1∶3∶2). Virus was titered as described below for HIV productive infection. PSC-macrophages were plated on a 24-well plate and spinoculated (1.5 h, 1800 *g*, 37°C) with NL4.3 BaL (Vpr-BlaM) or NL4.3 VSV-G (Vpr-BlaM) and incubated for 90 min-2 h at 37°C with 5% CO_2_. Cells were loaded with fluorogenic β-lactamase substrate CCF2-AM according to manufactures protocol and incubated overnight (GeneBLAzer™ *In Vivo* Detection Kit, 12578-134, Invitrogen). PSC-macrophages were detached by incubating with 0.25% trypsin (PAA) for 30 min-1 h at 37°C, and fixed at for 30 min 4°C in 4% formaldehyde in PBS. Fluorescence was measured using Dako Cyan™ (Beckman Coulter), and data was analysed using FlowJo software. Gates were set based on the fluorescent signal of uninfected samples loaded with CCF2-AM. Dot plots of a representative experiment are shown in Figure S1.

### qPCR of HIV-1 late reverse transcripts

To measure HIV-1 late reverse transcripts by qPCR, HIV-1 BaL viral stocks, obtained from *Gartner et al.* from the AIDS Research and Reference Reagent Program, Division of AIDS, NIAID, NIH and amplified in PBMCs for 12–21 days before harvesting [106], were treated with 100 µg/mL DNase I (Sigma). Macrophages were spinoculated with 500 µL DNase I treated HIV–1 BaL by centrifugation at 2000 *g* for 90 min at 37°C and incubation for an additional 30 min at 37°C. The inoculum was removed and replaced with fresh media. After incubating the cells for another 28 h in the incubator, media was removed and the cells (still on tissue culture plates) were stored at −20°C or used directly to extract DNA. DNA was extracted using DNeasy Blood and Tissue Kit (Qiagen) according to the manufacturer's instructions, except PBS and proteinase K were directly added onto the cells on the plates and cells were harvested from plates by pipetting up and down. DNA was eluted in AE buffer, supplied with the kit and used in a Taqman real-time qPCR reaction using Brilliant QPCR Core Reagent Kit (Agilent Technologies) with the following primers and probe. Forward primer 5′→3′: TGGGTTATGAACTCCATCCTGAT. Reverse primer 5′→3′: TGTCATTGACAGTCCAGCTGTC. Probe 5′→3′: FAM-TTTCTGGCAGCACTATAGGCTGTACTGTCCATT-TAMRA. The primer set used detects a conserved HIV-1 cDNA sequence, corresponding to a 81-bp fragment from the HIV-1 reverse transcriptase gene (*pol*). Reactions were set up using BrilliantqPCR Mastermix (Agilent Technologies), 375 nM forward primer, 375 nM reverse primer, 125 nM probe, 300 nM reference dye and ∼80 ng DNA from infected cells. Standards were prepared in duplicate using the pNL4.3.Luc.R-E- HIV-1 backbone plasmid diluted ranging from 10 to 1×10^7^ copies. To allow normalisation of the number of copies of HIV-1 DNA to the number of cells, β-actin DNA was quantified (β-actin control kit, Eurogentec) in parallel, according to the manufacturer's instructions. β-actin standards were prepared using human Xsomal genomic DNA (Eurogentec) at 10 fold dilutions ranging from 60 ng to 0.006 ng. The fast qPCR programme was as follows: 95°C for 3 min, and 40 cycles of 95°C for 5 sec, 60°C for 10 sec, and performed on an Applied Biosystems StepOne Plus Real Time PCR machine, with StepOne software.

### HIV-1 productive infection

Replication-defective pseudotyped HIV-1 was used to quantitate the stages of the viral life cycle up to and including integration into the genome of the target cell. HIV-1 pseudovirus Envelope proteins (HIV-1 JRFL, obtained from the NIH/AIDS reagents programme and the control envelope protein VSV-G) were expressed from the vector pcDNA3.1/Zeo (Invitrogen). For production of pseudotyped virus capable of a single round of replication, 293T cells were transfected as described previously [22], except the following ratios of plasmid were used: pNL4.3.Luc.R-E- (2.8 µg per well of 6-well plate) and pEnv or pVSV-G (0.7 µg per well of 6-well plate) in the ratio of 4∶1. To titer the virus, and also the virus used in the virion fusion assay (which contained the same luciferase gene) macrophages were spinoculated in 96-well plates with 100 µL of pseudotyped virus at 2000 *g* for 90 min at 37°C. Cells were incubated for 2 h in the incubator, before the virus inoculum was removed and replaced with 200 µL of medium. Three days later cells were lysed with ONE-Glo™ luciferase substrate (Promega) according to manufacturer's protocol. Luciferase expression from the viral genome was quantified using SpectraMax M5 with SoftmaxPro version 5 software. For experiments, equivalent, saturating titers of virus were used for both the fusion and productive infection assays.

### Statistical analysis

All data are represented as mean ±SEM of independent biological replicates is shown and statistical analysis was carried out with GraphPad Prism software. Statistical significance was reported as ns P>0.05; * P≤0.05; **P≤0.01; *** P≤0.001; **** P≤0.0001. For comparisons of means from multiple groups against one control group the one-way ANOVA using the Holm-Šídák post-test for multiple comparisons, with alpha  = 5.000%.

## Supporting Information

Figure S1 **HIV-1 viral fusion assay in PSC-macrophages.** A) Representative dot plots of transgenic PSC-macrophages infected with HIV-1 NL4.3 (BlaM-Vpr) and VSV-G NL4.3 (BlaM-Vpr) using the BlaM assay. Gates were set based on the fluorescent signal of uninfected samples loaded with the BlaM substrate.
(TIFF)

Figure S2 **Flow Cytometric screening test for Detergent Resistant surface antigens in macrophages.** A) To determine the optimal concentration of Triton X-100 (TX-100) that solubilises proteins that localize outside detergent resistant membranes (such as CD45), but does not affect detergent resistant proteins (such as CD14) in PSC-macrophages, PSC-macrophages were labelled with anti-CD14 and anti-CD45 or left unlabelled and treated with PBS or 0.025%, 0.030%, 0.040% cold TX-100 for 4 min. Fluorescence histograms of unlabelled (shaded grey) or anti-CD14 and anti-CD45 labelled PSC-macrophages after 4 min treatment with PBS (black line) or with 0.025% (purple line); 0.030% (red line); 0.040% (blue line) cold TX-100. B) Flow Cytometric Detergent Resistance (FCDR) index of unlabelled (open circles) CD14 (solid circles) and CD45 (solid squares) on PSC-macrophages, plotted against the detergent concentrations (0.025%, 0.030%, 0.040%). A cut-off of 0.45 is used to distinguish between proteins associated with the DRM and those found outside the DRM.
(TIFF)

Figure S3 **Expression of chimeric CD4 in PSC-macrophages.** A) To detect integration of lentiviral vectors, DNA was isolated from MDM derived from PSC infected with lentiviral vectors expressing shCD4 and chimeric CD4 (CD4_WT_, CD4_P-_, CD4_R-_, CD4_P-R-_) or from PSC infected with a lentiviral vector expressing a control hairpin (shCNTRL) and analysed by qPCR using specific primers. Symbols represent the mean number of copies ±SEM from technical replicates (n = 3) using pooled DNA from three independent experiments of chimeric CD4 relative to the CD4_WT_ group. B) To detect chimeric CD4 transcripts, RNA was isolated from transgenic PSC-macrophages and analysed by RT-qPCR using specific primers. Symbols represent the relative mean number of copies of transgenic chimeric CD4 mRNA relative to the CD4_WT_ group ±SEM of technical replicates (n = 3) using pooled RNA from three independent experiments. C) To detect protein expression of CD4, transgenic PSC-macrophages lysates were analysed by western blotting using anti-CD4 antibody. GAPDH, a loading control, was detected using anti-GAPDH antibody. Representative blot is shown. D) Protein levels were measured with Odyssey software (Li-COR) and CD4 expression was normalised to GAPDH expression. Symbols represent normalised CD4 expression, relative to the PSC-macrophages control group (shown in Figure 4) of three independent experiments ±SEM (n = 3).
(TIFF)

Figure S4 **Surface expression of chimeric CD4 in PSC-macrophages.** A) PSC-macrophages were tested for surface CD4 expression by flow cytometry using three different clones of anti-CD4 antibodies. Representative histogram showing CD4 surface staining with mAb clone 11830 (red/brown line, recognises both endogenous human and chimeric CD4) is shown in Figure 6E. Staining with mAb clone OKT4 (blue line, left panel, recognises endogenous human CD4 only); or with mAb clone OX-68 (orange line, right panel, recognises chimeric CD4 only). All lines are depicted compared to the matched isotype control (shaded gray). Representation of the binding site of the antibodies on human and chimeric CD4 are shown at the bottom. B) Quantification of endogenous human CD4 expression relative to the PSC-macrophages control group, using mAb OKT4 (n = 4). C) Quantification of chimeric CD4 relative to the PSC-macrophages CD4_WT_ group, using mAB OX68 (n = 6). D) Quantification of endogenous human CD4 and chimeric CD4 expression relative to the PSC-macrophages control group, using mAb 11830 (n = 7). B–D) The symbols reflect the relative ratio of the geometric mean fluorescence intensity (MFI) over the isotype control ±SEM.
(TIFF)
