# Supplementary figures and images for: The Productive Entry Pathway of HIV-1 in Macrophages Is Dependent on Endocytosis through Lipid Rafts Containing CD4
Source: PLoS One. 2014 Jan 22;9(1):e86071. doi: 10.1371/journal.pone.0086071 (PMC3899108; doi:10.1371/journal.pone.0086071)

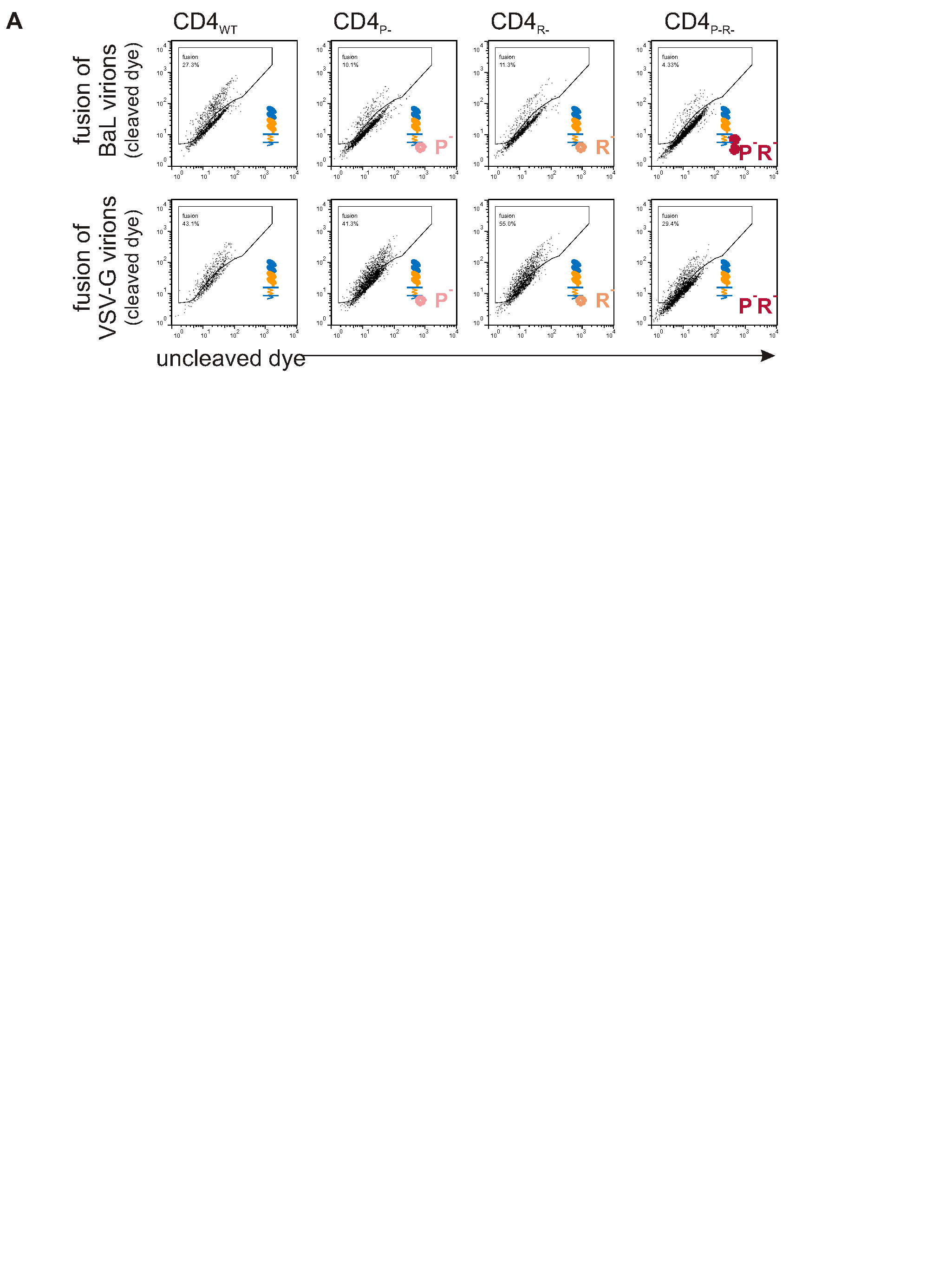

Supplement: Figure S1 — HIV-1 viral fusion assay in PSC-macrophages. A) Representative dot plots of transgenic PSC-macrophages infected with HIV-1 NL4.3 (BlaM-Vpr) and VSV-G NL4.3 (BlaM-Vpr) using the BlaM assay. Gates were set based on the fluorescent signal of uninfected samples loaded with the BlaM substrate. (TIFF) [file pone.0086071.s001.tiff]

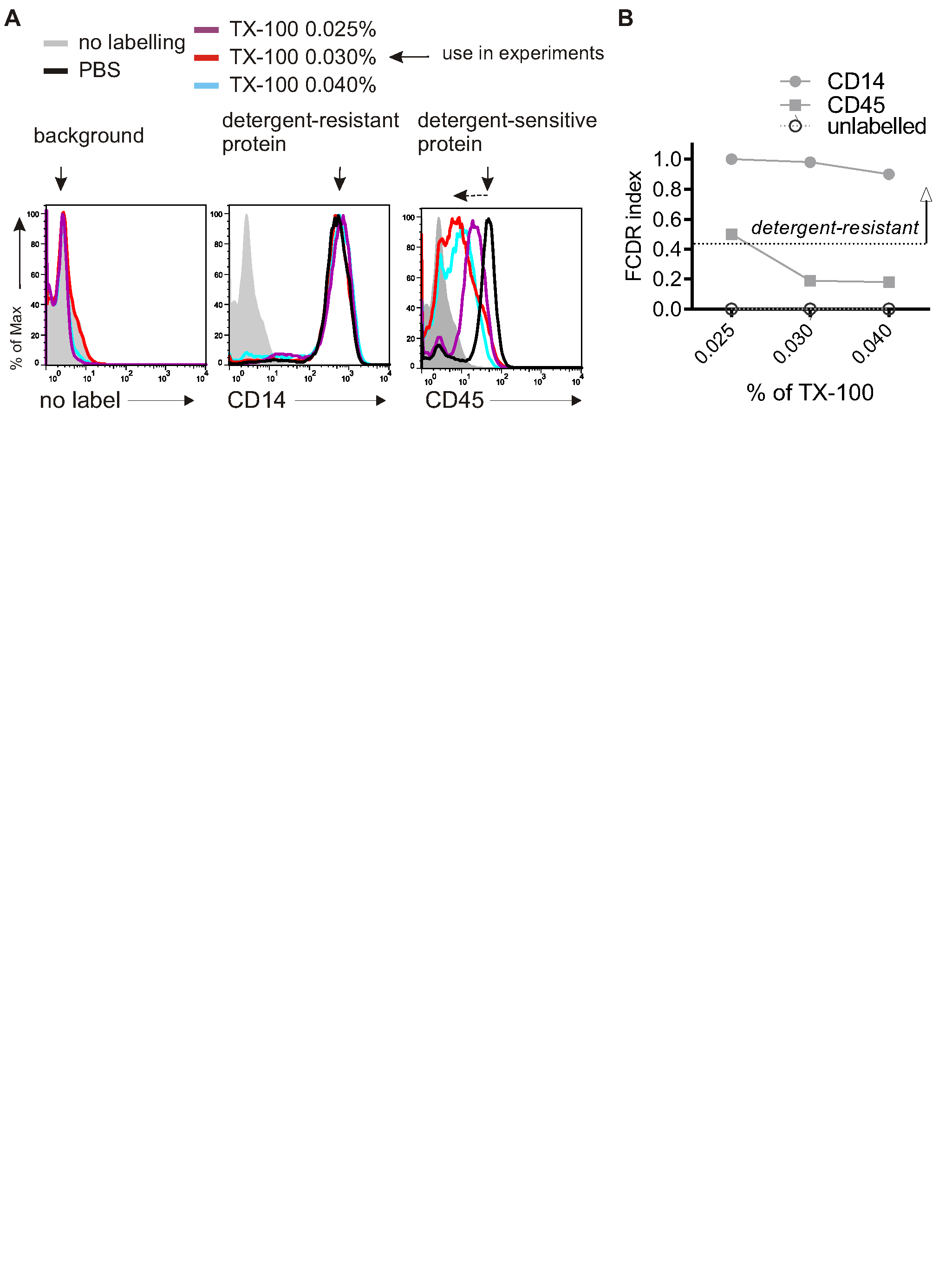

Supplement: Figure S2 — Flow Cytometric screening test for Detergent Resistant surface antigens in macrophages. A) To determine the optimal concentration of Triton X-100 (TX-100) that solubilises proteins that localize outside detergent resistant membranes (such as CD45), but does not affect detergent resistant proteins (such as CD14) in PSC-macrophages, PSC-macrophages were labelled with anti-CD14 and anti-CD45 or left unlabelled and treated with PBS or 0.025%, 0.030%, 0.040% cold TX-100 for 4 min. Fluorescence histograms of unlabelled (shaded grey) or anti-CD14 and anti-CD45 labelled PSC-macrophages after 4 min treatment with PBS (black line) or with 0.025% (purple line); 0.030% (red line); 0.040% (blue line) cold TX-100. B) Flow Cytometric Detergent Resistance (FCDR) index of unlabelled (open circles) CD14 (solid circles) and CD45 (solid squares) on PSC-macrophages, plotted against the detergent concentrations (0.025%, 0.030%, 0.040%). A cut-off of 0.45 is used to distinguish between proteins associated with the DRM and those found outside the DRM. (TIFF) [file pone.0086071.s002.tiff]

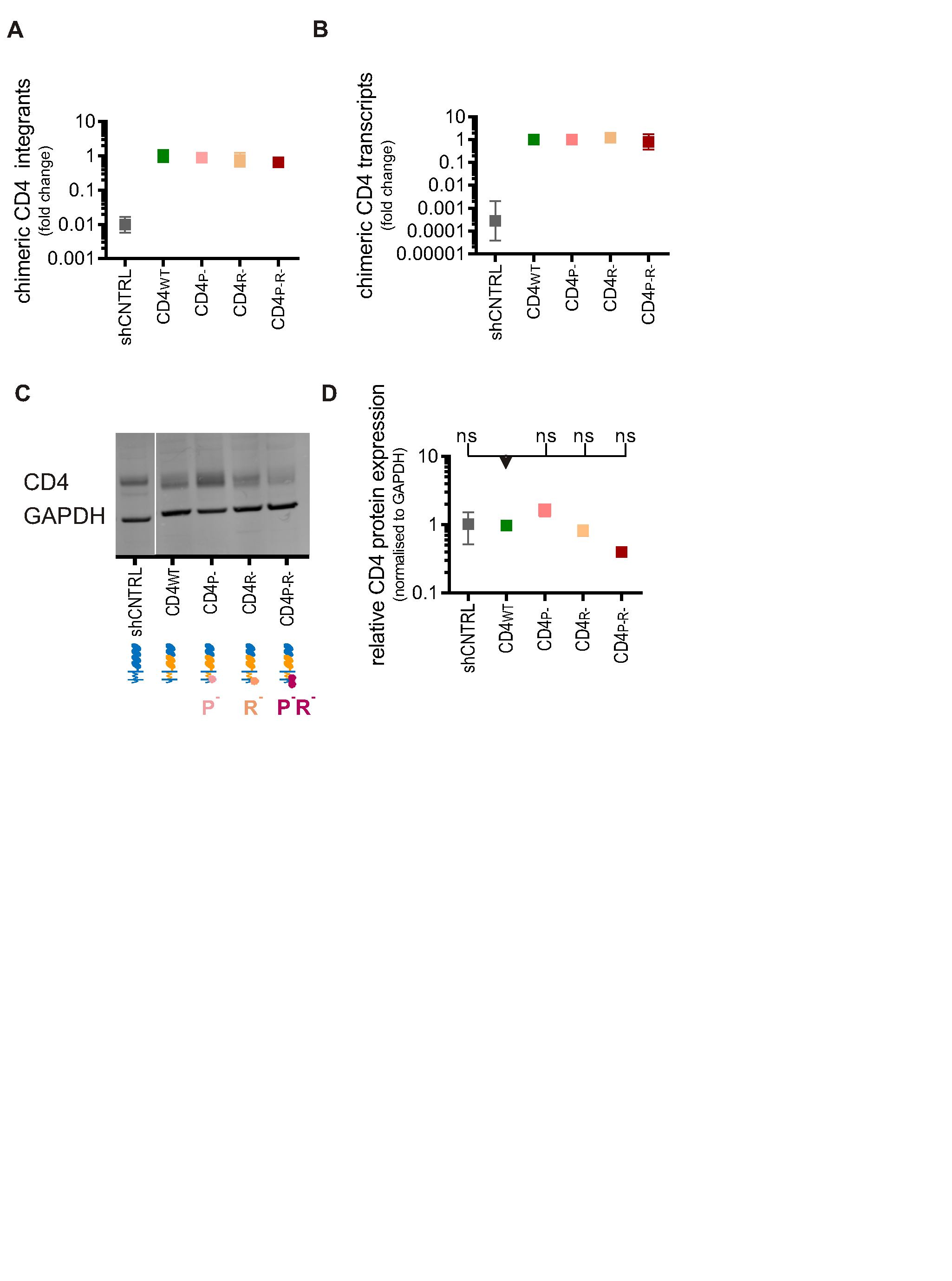

Supplement: Figure S3 — Expression of chimeric CD4 in PSC-macrophages. A) To detect integration of lentiviral vectors, DNA was isolated from MDM derived from PSC infected with lentiviral vectors expressing shCD4 and chimeric CD4 (CD4WT, CD4P-, CD4R-, CD4P-R-) or from PSC infected with a lentiviral vector expressing a control hairpin (shCNTRL) and analysed by qPCR using specific primers. Symbols represent the mean number of copies ±SEM from technical replicates (n = 3) using pooled DNA from three independent experiments of chimeric CD4 relative to the CD4WT group. B) To detect chimeric CD4 transcripts, RNA was isolated from transgenic PSC-macrophages and analysed by RT-qPCR using specific primers. Symbols represent the relative mean number of copies of transgenic chimeric CD4 mRNA relative to the CD4WT group ±SEM of technical replicates (n = 3) using pooled RNA from three independent experiments. C) To detect protein expression of CD4, transgenic PSC-macrophages lysates were analysed by western blotting using anti-CD4 antibody. GAPDH, a loading control, was detected using anti-GAPDH antibody. Representative blot is shown. D) Protein levels were measured with Odyssey software (Li-COR) and CD4 expression was normalised to GAPDH expression. Symbols represent normalised CD4 expression, relative to the PSC-macrophages control group (shown in Figure 4) of three independent experiments ±SEM (n = 3). (TIFF) [file pone.0086071.s003.tiff]

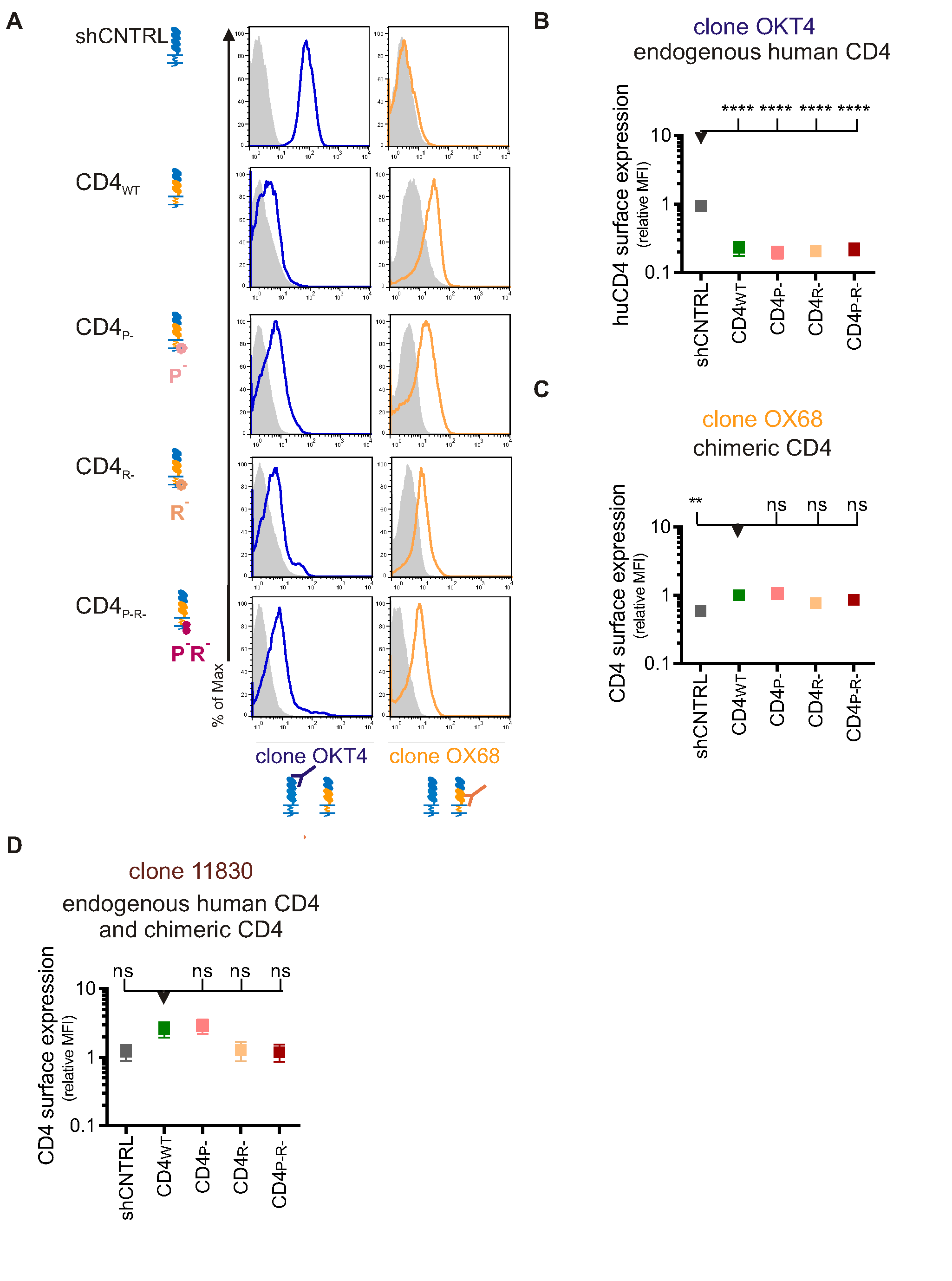

Supplement: Figure S4 — Surface expression of chimeric CD4 in PSC-macrophages. A) PSC-macrophages were tested for surface CD4 expression by flow cytometry using three different clones of anti-CD4 antibodies. Representative histogram showing CD4 surface staining with mAb clone 11830 (red/brown line, recognises both endogenous human and chimeric CD4) is shown in Figure 6E. Staining with mAb clone OKT4 (blue line, left panel, recognises endogenous human CD4 only); or with mAb clone OX-68 (orange line, right panel, recognises chimeric CD4 only). All lines are depicted compared to the matched isotype control (shaded gray). Representation of the binding site of the antibodies on human and chimeric CD4 are shown at the bottom. B) Quantification of endogenous human CD4 expression relative to the PSC-macrophages control group, using mAb OKT4 (n = 4). C) Quantification of chimeric CD4 relative to the PSC-macrophages CD4WT group, using mAB OX68 (n = 6). D) Quantification of endogenous human CD4 and chimeric CD4 expression relative to the PSC-macrophages control group, using mAb 11830 (n = 7). B–D) The symbols reflect the relative ratio of the geometric mean fluorescence intensity (MFI) over the isotype control ±SEM. (TIFF) [file pone.0086071.s004.tiff]
